# The white racial frame of public health discourses about racialized health differences and “disparities”: what it reveals about power and how it thwarts health equity

**DOI:** 10.3389/fpubh.2023.1187307

**Published:** 2023-09-26

**Authors:** Lisa Bowleg

**Affiliations:** ^1^Department of Psychological and Brain Sciences, The George Washington University, Washington, DC, United States; ^2^The Intersectionality Training Institute, Philadelphia, PA, United States

**Keywords:** white racial frame, framing public health discourses, health inequities, health equity, health disparities

## Abstract

Although several public health scholars have advocated for more clarity about concepts such as health disparities and health equity, attention to the framing of public health discourses about racialized health differences and “disparities” in the U.S., and what it reveals about power and the potential for achieving health equity, is surprisingly rare. Sociologist Joe Feagin, in his book, *The White Racial Frame: Centuries of Racial Framing and Counter-Framing* coined the term *white racial frame* to describe the predominantly white racialized worldview of majority white and white-oriented decisionmakers in everyday and institutional operations. Informed by insights from critical race theories about the white racial frame, white epistemological ignorance, and colorblind racism; critical perspectives on social class; Black feminist perspectives; framing; and critical discourse analysis, in this perspective I discuss: (1) the power of language and discourses; (2) the white racial frame of three common public health discourses — *health disparities*, “*race*,” and *social determinants of health* (SDOH); (3) the costs and consequences of the white racial frame for advancing health equity; and (4) the need for more counter and critical theoretical frames to inform discourses, and in turn research and political advocacy to advance health equity in the U.S.

## Introduction

There are children who read the dictionary for the sheer joy of it. I was one of them. Some of my fondest childhood memories find me reading my parents’ hardbound dictionary cover-to-cover as if it were a novel. Then, as now, I was enthralled by the multitude, diversity, and specificity of words within the English language. Now, informed by my work as a critical health equity researcher and scholar, my attention is acutely attuned to the language of public health in the U.S.: how discourses about racialized health differences and “disparities” are framed, what these frames reveal about power, and what this portends for health equity.

The issue is not simply one of political correctness or wokeness — simplistic but often effective derisions that function to silence serious dialogue about power and privilege, and when wielded by those with greater social power, exemplify precisely how power operates. Nor is it just about semantics. Rather, frames are important because they entail “the *select [ion of] some aspects of a perceived reality and mak [ing] them more salient in a communicating text, in such a way as to promote a particular problem definition, causal interpretation, moral evaluation, and/or treatment recommendation*” (italics in original) [(1), p. 52].

Advocacy to define and clarify language about terms such as *health disparities*, *health differences*, *health inequality*, *health inequity*, and *health equity* is hardly new (2–9). More than three decades have passed since health equity expert Margaret Whitehead authored “Concepts and Principles of Equity and Health” on behalf of the World Health Organization (WHO) (9). An essential primer on the lexicon on health equity, the WHO report defined health inequities as “differences in health which are not only unnecessary and avoidable but, in addition, are considered unfair and unjust” [(9), p. 220]. As with health differences between groups such as children and the older adults, or those who participate in high-risk sports compared with those who do not, not all health differences are unjust. But the racialized health inequities that are the focus of most health “disparities” research and programs in the U.S. — heart disease, cancer, preterm delivery, maternal mortality, HIV and COVID-19, for example — *are* avoidable by reasonable means, and thus by WHO’s definition, inequitable. That these same health differences are endemic and disproportionate in U.S. populations systematically marginalized and oppressed by structural racism and intersecting sexism, cissexism, heterosexism, ableism, and classism (to name a few) underscores that exposure to structural racism and intersectional discrimination are fundamental causes of health inequities.

Yet, with a handful of notable exceptions (10–16), attention to what the framing of public health discourses about racialized health differences and “disparities” in the U.S. reveal about power is surprisingly rare. Framing offers important insights about how political and social power shape hegemonic ideologies in discourse (17). Enter the white racial frame. Coined by sociologist Joe Feagin [(18), p. 3], the white racial frame describes “an overarching white worldview that encompasses a *broad and persisting set of racial stereotypes, prejudices, ideologies, images, interpretations, and narratives, emotions, and reactions to language accents, as well as racialized inclinations to discriminate* (italics in original).

Informed by insights from critical race theories (13, 19–23), critical perspectives on class (24–27), Black feminist perspectives (28–30), and the literature on framing (1, 15, 17, 18) and critical discourse analysis (31, 32), I discuss the power of language and discourses; the white racial frame of three common public health discourses — *health disparities*, “*race*,” and *social determinants of health* (SDOH); the costs and consequences of the white racial frame for advancing health equity; and the need for more counter and critical theoretical frames to inform discourses, and in turn research and political advocacy to advance health equity in the U.S.

## The power of language

Language is powerful. Philosophers such as Paulo Freire (33), Frantz Fanon (34), and Michel Foucault (35), and many feminist scholars (36–39) have asserted that language is never neutral, but rather a mirror that reflects power. Freire in his groundbreaking book *Pedagogy of the Oppressed* noted, “the problems of language always involve ideological questions and along with them, questions of power” [(40), p. 132]. Foucault posited that power was implicated and transmitted, not just in social structures and politics, but in everyday discourses about constructs such as sexuality (35) and madness (41). Foucault’s work spawned Foucauldian Discourse Analysis, a branch of critical discourse analysis that asserts that language controls and shapes what we think, know, and think we can know; reflects powerful discursive systems (e.g., biomedical discourses); and influences what we consider possible for action or intervention (31, 32). As for feminist theory, a plethora of feminist scholars have lambasted sexism in the English language, noting how sexual derogations of women (e.g., mistress vs. master) or derivations from the masculine (e.g., princess, actress) mirror women’s lower social power compared with men’s (36–39).

As for the framing of public health discourses about health differences and “disparities,” British critical psychologist Ian Parker advises that “language is so structured to mirror power relations that often we can see no other ways of being, and it structures ideology so that it is difficult to speak both in and against it” [(32), p. xi]. To wit: the ubiquitous and mostly uncritical embrace of terms such as *health disparities, “race,” and social determinants of health* within U.S. public health. Because “discourses both facilitate and limit, enable and constrain what can be said (by whom, where, when),” [(32), p. xiii] the framing of discourses offers important insights into power. For example, the fact that many of the debates about how to define health disparity in the U.S. have centered around whether or not to include social justice (4, 6), the extent to which “disparities” are avoidable (7), or that the uptick of the term *health equity* within U.S. public health is relatively recent (4), evinces that “institutions … are structured around and reproduce power relations” [(32), p. 18]. As such, public health discourses and their frames are not neutral, but ideological and political (11, 12, 15). Political scientists Lynch and Perera [(15), p. 806], in an insightful comparative analysis of health equity frames in U.S., United Kingdom, and French government reports over a 30-year span assert that political frames about health inequality and equity function to show “who is to blame, who is responsible — and can be expected to shape the policies intended to address the problem.”

## The white racial frame of discourses about *health disparities*, “*race*,” and *SDOH*

In the context of the framing of public health discourses about racialized health differences and “disparities” in the U.S., the white racial frame encompasses:

Majority-white decisionmakers [who] include public health researchers and policymakers, medical educators and officials, hospital administrators, and insurance and pharmaceutical executives, as well as important medical personnel … [whose] racial framing and racialized actions have created, shaped, or maintained these health inequalities—and the health-related institutions imbedding racial framing and inequalities [(13), p. 8].

Although white racial frames relevant to three discourses — *health disparities*, “*race*,” and *SDOH* — are my focus here, many terms and phrases in the field reflect this frame; commonly used adjectival modifiers of racialized and other minoritized groups such as “vulnerable,” “at-risk,” or “high risk populations” come to mind. The power of absence is also noteworthy. Missing discourses signal what the field deems acceptable or appropriate to say or not. Take for example, the relative dearth of discourses about white privilege, oppressors, discriminators, class privilege, capitalism, class exploitation, class struggle, oppressed groups, victimized groups, enriched groups, advantaged groups, white supremacy, and racists.

### Health disparities

In the U.S., *health disparities* is the term that public health officials, researchers, policymakers, and political elites use most often. By contrast, countries such as the United Kingdom, France and Canada use the term *health inequalities* and often *use it* synonymously with the term *health inequities* (6, 7, 15). *Healthy People 2030* (42), a decennial key initiative of the U.S. Department of Health and Human Services defines health disparities as:

A particular type of health difference that is closely linked with social, economic, and/or environmental disadvantage. Health disparities adversely affect groups of people who have systematically experienced greater obstacles to health based on their racial or ethnic group; religion; socioeconomic status; gender; age; mental health; cognitive, sensory, or physical disability; sexual orientation or gender identity; geographic location; or other characteristics historically linked to discrimination or exclusion.

From a critical discourse analysis perspective, this definition is hard at work in terms of what it is saying and taking pains *not* to say. Among its notable discursive features are the positioning of discrimination or exclusion last, the framing of key verbs in the past participle (e.g., “linked,” “experienced”), the double inference of correlation (“closely linked” and “historically linked)” limiting inferences about causality, the passive positioning of health disparities as something “systematically experienced” by certain groups located at certain demographic positions (e.g., racial or ethnic group, gender, age) rather than the active attribution of the disparities to “discrimination or exclusion,” and the use of the adverb “historically” which discursively places such discrimination or exclusion in historical (i.e., the past) rather than contemporaneous context.

At the heart of this definition and that of many other U.S. government reports on health differences and “disparities” are theories about “… the origin of disease and ill health, and more specifically about the origin of inequality in their distribution, and one about the relationship between scientific knowledge and political action” [(43), p. 64]. The federal government’s investments in selecting what can and should be said — *health disparities* but not *health inequalities* or *health inequities* — aptly spotlights the white racial frame. There are at least three instantiations of this: (1) postpositivist axiological assumptions that connote that a commitment to social justice is inappropriate, negative, or at best, optional; (2) concerns about ideological or emotional backlash; and (3) postpositivist epistemological and methodological concerns. As an example of the first and second, Braveman speculates that the National Institutes of Health (NIH) adopted its prior (not current) definition of health disparity as “differences in prevalence, incidence, or severity of disease among different population groups,” in an effort to “avoid ideological controversy [by using a definition that was] purely technical, free of explicit or implicit values and thus would not provoke the backlash that often comes with actions that invoke social justice” [(4), p. 596]. The white racial frame thus functions as a “bureaucratization of oppression” within U.S. institutions, including the field of public health; one of racialized understandings that deem the needs, values, and concerns of white people to be superior and normal, and those of “people of color as groups to be generally of less social, economic, and political consequence” [(18), p. 141].

Even though many contemporary definitions of health “disparity” mention social injustice in some form or fashion, concerns about emotional backlash endure. Braveman, for example, eschews advocacy to substitute the term *health inequities* for *health disparities* with the argument that:

… “inequity” is highly charged emotionally; repeatedly using “inequities” would detract from the potential power of the term. For this reason, too, a judicious, sparing use of “inequities” seems advisable, so that it keeps its force, rather than substituting it for “disparities” [(4), p. 596].

This prompts the question: for whom is the word *health inequity* highly emotionally charged? It is unfathomable that it would discomfort the groups marginalized by structural racism and interlocking oppressive systems (e.g., sexism, heterosexism, *and* cisgenderism); those who, because of intersectional social-structural discrimination, bear the inequitable and disproportionate brunt of pain, suffering, and excess morbidity and mortality. Thus, emblematic of the white racial frame, concerns about “white fragility” (44), keeping white people comfortable, and reducing the risk of white people’s political and ideological backlash, surpass commitments to social justice and health equity for racialized and minoritized groups.

The notion that structural racism is a fundamental cause of health inequities is well-documented in the vast theoretical and empirical literature on discrimination and health (45–51). Underscoring yet another example of the white racial frame of discourses about health “disparities” is the dominance of postpositivist assumptions about causation. Braveman avers that the term health “disparity” is a tremendously useful concept, because although it implies that there is something [morally or ethically] suspect about an observed difference, it does not require proof of causation” that is, “… the differences in health are caused by social injustice” [boldface and underline in original; (4), p. 596]. The focus on causation is problematic, Braveman argues, because it could necessitate: “… [a] need to provide, for each difference deemed a disparity according to this definition, that the difference was caused by unfair and unjust policies, conditions or actions.” She adds:

It is exceedingly difficult to provide causation, i.e., that X (unjust policies etc.) caused Y (a difference in health). This is particularly true given the field of medicine’s strong tendency to reject causal evidence if it does not come from a randomized clinical trial (RCT). Furthermore, many, perhaps most, of our hypotheses about the role of social factors influencing health do not lend themselves to RCTs; the effects of the social factors often play out across complex pathways manifesting in health outcomes only after decades or sometimes generations [(4), p. 596].

In seeking to sidestep attacks about causation, Braveman invokes the biomedical discourse, *the* powerful and dominant discourse of medicine and science, and epistemic credibility. In this postpositivist formulation, the RCT is the apotheosis for what constitutes “evidence.” Other sources of knowledge or “evidence” such as narratives from enslaved or formerly enslaved people about their lives, a plethora of empirical research on structural racism and negative health outcomes (52, 53), countless peer-reviewed articles and books on the history of scientific and medical racism (54–59), and personal narratives of people from intersectionally marginalized groups (see for example, Black lesbian feminist, Audre Lorde’s poignant writing about intersectional discrimination and health in *The Cancer Journals* (60) and *A Burst of Light: Living with Cancer*) (61), collectively lack the epistemic credibility to provide evidence that social injustice is a fundamental cause of health inequities. It reflects a Eurocentric masculinist paradigm; one that valorizes what Black feminist sociologist Patricia Hill Collins in *Black Feminist Thought* has defined as “the institutions, paradigms, and other elements of the knowledge validation procedure controlled by elite white men” [(28), p, 203]. This paradigm also renders knowledge from marginalized groups such as Black women to be simply subjective and irrelevant. The white racial frame of the biomedical discourse about health differences thus constrains what can be known about social injustice and health, and also who can know it. It functions as a *White logic*, “a context in which White supremacy has defined the techniques and processes of reasoning about social facts … [and] assumes a historical posture that grants eternal objectivity to the views of elite Whites and condemns the views of non-Whites to perpetual subjectivity” [(20), p. 17].

Not all health differences are inequalities or inequities. But, to frame widely known and observable health differences that are foundationally and patently the result of structural racism as “disparities” rather than inequalities or inequities in service of quelling white emotional, political or ideological backlash, is to engage in racialized epistemological ignorance (62, 63). The white racial frame bolsters white supremacy by making salient discourses that imply that racialized differences in health are natural, privileges evidence of causation, and seeks to avoid white people’s emotional or political discomfort. In so doing, the frame distracts attention away from the historical, political, and structural determinants of health inequities; conceals the moral and ethical urgency to reduce and eliminate inequities; and obscures the imagination and political will needed to develop, implement, and evaluate effective and high-impact political and structural interventions.

## The racecraft of “race”

Despite widespread scientific consensus that no genetic or biological basis for “race” exists (55, 64–66), and growing advocacy to eschew “race” as scientifically or biologically meaningful (5, 55, 67–71), public health discourses that invoke “race” are ubiquitous in the U.S. The history of white supremacy and racism in science, and the dogged conviction of white scientists (and politicians) across centuries and disciplines to establish white people as superior to justify racial stratification (72, 73), is amply documented. Thus, the tenacity of public health discourses about health differences and “disparities” by “race” reflect a white racial frame and colorblind racism that locates the problem of health inequities within the bodies of racial/ethnic minoritized people rather than structural racism. Take for example the footnote that accompanies the Centers for Disease Control and Prevention’s “Risk for COVID-19 Infection, Hospitalization, and Death By Race/Ethnicity” table that documents racialized inequities in COVID-19 (74). The table shows that compared with white non-Latina/x/o people, American Indian, Black, and Latino people have disproportionately higher rates of hospitalization (i.e., 2.5, 2.1, and 1.9 times, respectively) and death (i.e., 2.1, 1.6, and 1.7 times, respectively). A footnote to the table declares that “race and ethnicity are risk markers for other underlying conditions that affect health, including socioeconomic status, access to health care, and exposure to the virus related to occupation, e.g., frontline, essential, and critical infrastructure workers.”

In this discursive construction, racism disappears as the culprit that explains these differences in socioeconomic status, access to health care, and occupational exposure, leaving only “race.” It is the epitomic power play of the white racial frame. In their book *Racecraft*: *The Soul of Inequality in American Life,* sociologist Karen E. Fields and historian Barbara J. Fields (75) illustrate the mythical and magical thinking that crafts “race” as scientifically meaningful and “transforms racism into race.” They write:

Racism always takes for granted the objective reality of race … The shorthand transforms racism, something an aggressor does, into race, something the target is, in a sleight of hand that is easy to miss. Consider the statement “Black southerners were segregated because of their skin color” — a perfectly natural sentence to the ears of most Americans, who tend to overlook its weird causality. But in that sentence, segregation disappears as the doing of segregationists, and then, in a puff of smoke — puff — reappears as a trait of only one part of the segregated whole” [(75), p. 17].

So omnipresent is this racecraft in public health discourses about racialized differences and “disparities” that is it both striking and anomalous to find declarations such as “Disparities in COVID-19 outcomes likely stem from structural racism on many levels” [(76), p. 1630], a key conclusion of analyses of a nationally representative study on racial/ethnic “disparities” in COVID-19 risk, employment and household composition. Abandoning the word “race” does not obviate the need to collect data on *racialized* or racially *minoritized* data however; such data are vital to assess and document racialized health inequities (51, 77). Acknowledging the irony of using data on “race” to document racialized health inequities such as COVID-19, Krieger (51) advises researchers to explicitly justify how they have conceptualized and categorized racial groups, and to analyze individual-level racialized data within the context of racialized social inequities.

The intensive focus on “race” in public health discourses about health inequity also obscures the foundational role of social class — traditionally conceptualized in terms of occupation, education, and income — as a key determinant of health in the U.S. (24, 26). In this regard, the white racial frame of public health discourses about health inequity is also a homogenizing discourse about whiteness. It elides critical health inequities among white people at the intersection of social class, obscuring the existence of a pronounced socioeconomic health gradient whereby the health of rich people is markedly better than that of middle class people, whose health is markedly better than that of working class people (78). For many white people in the U.S. — those who “do not see color” and revel in being colorblind, those who relish the white savior role, and those who are either subtlety, aversively, or explicitly racist — the white racial fame is also expedient. At one end of the continuum, the white racial frame provides solace based on the perception that if only everyone (like they of course do) ate healthy foods, exercised, visited physicians regularly, and abstained from “risky” sex or certain drugs, there would be no health disparities or inequities. At the other end, it animates and stokes racial resentment and grievance, prompting many conservative white people to vote against policies and legislation that would improve their health and well-being; thereby literally “dying of whiteness” (79).

Bolstering a need for more intersectional perspectives in public health (80), the almost exclusive single-axis (i.e., “race” solely) focus of white racially framed public health discourses about racialized health differences on the single-axis of “race” rather than at the intersection of racial group *and* class, functions to distract important and necessary attention from social class as a core determinant of health inequalities in the U.S. for all, including white people.

The power of institutional discourses about “race,” health differences, and health disparities also influence the allocation of resources to fund research on “race.” Friedman and Lee (81) document how the U.S. Congress’ passage of the NIH Revitalization Act of 1993 which mandated the inclusion of “women and [racial/ethnic] minorities” [*sic*] be included in all research with human participants, spurred an increase in National Cancer Institute grants focused on “race” or ethnicity as a specific research focus (52% pre-vs. 64% post-mandate). Yet, only a handful of the 72 grants explained, hypothesized or theorized the nature of racial or ethnic differences (18%), or explained or theorized the importance of “race” or ethnicity (30%). Friedman and Lee’s analyses of the peer-reviewed articles based on these grants found that although 91% of the articles mentioned “race,” only a handful (28%) cited prior knowledge about racial differences or defined “race” or ethnicity (31%). Nonetheless, most of the articles (63%) reported analyses with “race” or ethnicity as a variable or stratified their variables by “race.”

That most of the research treated “race” as scientifically meaningful and axiomatic underscores the formidable power of the white racial frame in institutional discourses about racial and ethnic differences in the U.S., and the federal government’s considerable investment in the production of knowledge about racial differences in health research. Grant funding in turn shapes the types of research questions that researchers deem important to investigate, the theoretical frameworks they use — historically, individual-level biomedical and psychosocial theories, almost exclusively (82) — and the methods they employ — traditionally, quantitative methods and experiments, with the RCT established as the gold standard.

Emblematic of how power works to maintain the status quo, one instantiation of the white racial frame is social and behavioral science fields, public health among them, that prioritize research, research, and ever more research to document health inequities, but evince comparatively little interest or investment in developing or evaluating multilevel, structural, or political interventions that could transform intersecting systems of structural oppression, and in turn reduce or eliminate health inequities. The power of the white racial frame about “racial” health differences and disparities is also reflected in the ways that predominantly white universities and other institutions valorize and reward (mostly white) people who write grant applications, conduct research, and write and publish and peer-reviewed articles on “race” and health “disparities,” but disparage approaches that examine, criticize, and challenge the legal and political determinants of health inequities as too political, not research, or “just” activism.

## Social determinants of health

Social determinants of health (SDOH) refer to “the non-medical factors that influence health outcomes. They are the conditions in which people are born, grow, live, and age, and the wider set of forces shaping the conditions of daily life” (83). Used in the U.S. since 2010, the SDOH framework highlights five key domains beyond the individual-level that are related to health inequities: economic stability, education access and quality, social and community context, health care access and quality, and neighborhood and built environment (84). Underscoring the power of the white racial frame in public health discourses, structural racism is notably absent from the conventional SDOH framework (85). Instead, “discrimination” is listed under social and community context, in conjunction with civic participation and incarceration. In line with her insightful argument that structural racism has the greatest influence on all five key areas of the SDOH, Professor of Law Ruqaiijah Year by (85) has proposed a reconfiguration of the SDOH framework that prioritizes structural racism and intersectional structural discrimination (e.g., sexism, ableism, heterosexism) as the root cause of health inequities.

That structural racism has been absent from SDOH frameworks is noteworthy, but not surprising. Its absence is yet another example of the white racial frame and color-blind racism, the ideology that racialized social inequities are the consequence of nonracial factors such as market dynamics, naturally occurring phenomena and the cultural limitations of Black and other racial/ethnic minority people (19). As an ideology, public health discourses about the “social” determinants of health rather than the structural or political determinants of health function to obscure and minimize the role of structural determinants, particularly the historical and ongoing legacies of structural racism (e.g., slavery, the Black codes, Jim Crow, de jure racial segregation). These discourses also function to occlude the considerable influence of political determinants of health and health inequities in the U.S. (86–88). Countries like the U.S. where market capitalism dominates, welfare benefits are provided based on means and need (vs. citizenship or worker’s rights), income differentials are greatest, and public health expenditures are low, have the largest health inequities on indicators such as infant mortality (87). Other political determinants in the U.S. include the absence of a social protection floor (e.g., universal health care, basic income) (88), the “American health paradox” whereby the U.S. spends more of its Gross Domestic Product on health care than any other country but has the worst health outcomes of the world’s 37 high-income democracies (89–91), and a health care system characterized by the “commodification of suffering” of patients deemed “higher risk” (10), and “unchecked greed … and glorification of profit” [(92), p. 630].

Although the white racial frame of U.S. public health discourses has been my primary focus, it bears noting that such discourses are not U.S.-limited. Globally, the white racial frame has its analogs in a host of colonialized and imperialist worldviews that implicitly position indigenous people and people of color — racial/ethnic “minorities” in the U.S., but majorities, globally speaking — as “Other,” minoritized, and in turn, as primitive, ignorant, “risky,” “vulnerable,” and/or in desperate need of Western (read white) salvation. Beyond the U.S., colonialist and imperialist white racial frames ignore the racist systems of structural oppression (93) that seed and sustain global health inequities. Consider WHO’s (94) reflection on structural racism and health. It notes “Across the globe, Indigenous Peoples as well as people of African descent, Roma and other ethnic minorities experience stigma, racism and racial discrimination.” This neo-colonialist construction implicitly minoritizes the global majority while neglecting the systems of oppression rooted in slavery, colonialism, and imperialism, that are precursors to health inequities for historically oppressed groups. Similarly, structural racism is nowhere to be found on WHO’s (83) list of 10 SDOH examples that can positively and negatively shape health equity. “Social inclusion and non-discrimination” made the list, a far cry from the histories of slavery and colonialism that characterize most of the countries and regions with pervasive health inequities.

It is the ultimate checkmate of white supremacy, that the white racial framing of SDOH functions to implicate racialized and other minoritized groups themselves, and “social” factors of their behaviors, cultures, lifestyles, and neighborhoods as core determinants of health inequalities and inequities. Likely echoing criticisms of “social” as the modifier in the SDOH framework, there appears to be a small but growing increase in theoretical and empirical articles using terms such as “social-structural,” “social and structural,” or “structural” as adjective modifiers of determinants of health.

## Discussion

The white racial frame of public health discourses about racialized health differences and “disparity” is inimical to the goal of advancing health equity. It orients the field towards the fiction that “disparities” are not foundationally the consequence of longstanding, systemic and structured power relations; fosters the myth that “race” is scientifically meaningful; that racialized differences in health are primarily the result of biology, genetics, lifestyle choices, cultural deficits and/or individual-level cognitions and behaviors; and perpetuates the fallacy that primarily individual-level social, rather than political and structural determinants, are the fundamental causes of health inequity. Collectively, the white frame of public health discourses about health differences and “disparity” distract attention from “the heart of the matter: power … and that the science of health inequities can no more shy away from this question [of power] than can physicists ignore gravity or physicians ignore pain” [(86), p. 169].

In one of her most famous and searing essays, “The Master’s Tools Will Never Dismantle the Master’s House,” Audre Lorde [(29), p. 111] posed the provocative question: “What does it mean when the tools of a racist patriarchy are used to examine the fruits of that same patriarchy?” Lorde answered: “… it means that only the most narrow perimeters of change are possible and allowable” [(29), p. 111]. As a master’s tool, the white racial frame not only limits and constrains what can be said (by whom, where, and when) (32), but more fundamentally limits and constrains the types of questions the field deems important to ask about inequality and inequity, the methods used to examine them, and the solutions and interventions deemed possible to solve the problem. This too, is emblematic of power. In a country where the overwhelming majority of politicians, policymakers, and researchers are white and often middle or upper-class, the questions and solutions about health deemed important to fund and research are typically those that converge with the interests of the powerful, not the oppressed.

This notwithstanding, there is cause for cautious optimism about the framing of public health discourses about health inequality, inequity, and equity. Foucault reminds that although “… discourses transmit and produce power, discourses also undermine and expose power, rendering it fragile and possible to thwart” [(35), pp. 100–101]. The field of public health thus has opportunities to expand the perimeters of change beyond the narrow confines of the white racial frame by broadening its frames to include more political (26, 86, 95), critical race theory (21–23), intersectional (80, 96), critical public health (97), legal epidemiology (98, 99), ecosocial (100, 101), human rights (102, 103), and structural racism (45, 46, 48, 51) frames. Historically peripheral to health inequality and inequity discourses and approaches in the U.S., these counter-frames offer vital and essential epistemic resistance by challenging “the unexamined assumptions of epistemic power [and] standard approaches to methodology [that] underemphasize the significance of power relations within intellectual inquiry” [(104), p. 143].

And yet, having more counter reframes and critically informed discourses will not reduce or eliminate health inequities. The field of public health cannot (and will not) theorize and research itself out of this “wicked problem.” Moreover, because most of the key determinants of health inequality and inequity are political and structural, “reducing health inequalities may be even more politically difficult than researchers generally think” [(15), p. 804]. To dismantle health inequalities and inequities — not just research or document them — the field will need to pay greater attention to the political and structural determinants of health inequity, more multidisciplinary collaborations and approaches (e.g., sociology, political science, social and legal epidemiology), more critical theoretical frameworks, more attention to critical praxis, more community-engaged and led research — the Multicultural AIDS Coalition (MAC), Inc. (105, 106) a nonprofit organization with the mission of mobilizing communities of color to end the HIV epidemic, and SisterLove (107, 108), the first women’s HIV/AIDS and reproductive justice organization in the southeastern U.S. are exemplars, — more grassroots mobilization and political advocacy for more equitable laws and policies, and more multilevel (e.g., individual, community and structural-level) interventions. White racially framed public health discourses about “disparity,” “race” and “social” determinants of health thwart the goal of advancing health equity in the U.S. These are tools of the master; they will not dismantle the master’s house.

## Data availability statement

The original contributions presented in the study are included in the article/supplementary material, further inquiries can be directed to the corresponding author.

## Author contributions

The author confirms being the sole contributor of this work and has approved it for publication.

## Funding

This work was supported by the DC Center for AIDS Research and the Department of Psychological and Brain Sciences, The George Washington University, Washington, DC.

## Conflict of interest

The author declares that the research was conducted in the absence of any commercial or financial relationships that could be construed as a potential conflict of interest.

## Publisher’s note

All claims expressed in this article are solely those of the authors and do not necessarily represent those of their affiliated organizations, or those of the publisher, the editors and the reviewers. Any product that may be evaluated in this article, or claim that may be made by its manufacturer, is not guaranteed or endorsed by the publisher.
